# Kidney biopsy in very elderly patients: indications, therapeutic impact and complications

**DOI:** 10.1186/s12882-021-02559-9

**Published:** 2021-11-02

**Authors:** Mathilde Fedi, Mickaël Bobot, Julia Torrents, Pierre Gobert, Éric Magnant, Yannick Knefati, David Verhelst, Gaëtan Lebrun, Valérie Masson, Philippe Giaime, Julien Santini, Stanislas Bataille, Philippe Brunet, Bertrand Dussol, Stéphane Burtey, Julien Mancini, Laurent Daniel, Noémie Jourde-Chiche

**Affiliations:** 1 AP-HM, University Hospital of la Conception, Nephrology and Kidney Transplant Centre, Marseille, France; 2 Aix-Marseille Univ, C2VN, INSERM, INRAE, Marseille, France; 3 AP-HM, University Hospital of La Timone, Anatomical Pathology Laboratory, Marseille, France; 4 Rhône Durance Clinic, Medicine Department Avignon, Avignon, France; 5 Private Hospital of Provence, Nephrology Department, Aix-en-Provence, France; 6 Hospital of Sainte Musse, Nephrology Department, Toulon, France; 7 Hospital Général Henri Duffaut, Nephrology Department, Avignon, France; 8 Hospital of Aix en Provence, Nephrology Department, Aix-en-Provence, France; 9 Polyclinic “des fleurs”, Nephrology Department, Ollioules, France; 10 Phocaean Institute of Nephrology, Bouchard Clinic, ELSAN, Marseille, France; 11 Saint-Joseph Hospital, Nephrology Department, Marseille, France; 12 Aix Marseille Univ, APHM, Inserm, IRD, SESSTIM, University Hospital of la Timone, BIOSTIC Service, Marseille, France

**Keywords:** Kidney biopsy, Elderly patient, Advanced age, Acute kidney injury, Nephrotic syndrome, Glomerulonephritis, Pathology, Adverse events, Treatment, Survival

## Abstract

**Background:**

Few data is available on the risk/benefit balance of native kidney biopsy (KB) in very elderly patients.

**Methods:**

Multicenter retrospective cohort study in the Aix-Marseille area: the results of KB and medical charts of all patients over 85 years biopsied between January 2010 and December 2018 were reviewed.

**Results:**

104 patients were included. Median age was 87 years. Indications for KB were: acute kidney injury (AKI) in 69.2% of patients, nephrotic syndrome (NS) with AKI in 13.5%, NS without AKI in 12.5%, and proteinuria in 4.8%. Median serum creatinine was 262 μmol/L, 21% of patients required dialysis at the time of KB. Significant bleeding occurred in 7 (6.7%) patients, requiring blood cell transfusion in 4 (3.8%), and radiological embolization in 1 (1%). The most frequent pathological diagnoses were: non-diabetic glomerular diseases (29.8%, including pauci-immune crescentic glomerulonephritis in 9.6%), hypertensive nephropathy (27.9%), acute interstitial nephritis (16.3%), renal involvement of hematological malignancy (8.7%), and acute tubular necrosis (6.7%). After KB, 51 (49%) patients received a specific treatment: corticosteroids (41.3%), cyclophosphamide (6.7%), rituximab (6.7%), bortezomib (3.8%), other chemotherapies (3.8%). Median overall survival was 31 months.

**Conclusions:**

KB can reveal a diagnosis with therapeutic impact even in very elderly patients. Severe bleeding was not frequent in this cohort, but KB may have not been performed in more vulnerable patients.

## Introduction

As life expectancy increases, the proportion of elderly patients with renal diseases raises [1, 2]: the medical care of elderly patients is changing in developed countries [3]. The life expectancy in France in 2019 was 85.6 years for women and 79.7 years for men. The median age of dialysis initiation was 70.9 years in 2018 [4] and 12.9% of prevalent patients on chronic dialysis were older than 85 years.

Kidney biopsy (KB) is an invasive diagnostic procedure aimed at identifying kidney diseases and guiding treatment to prevent progression to chronic kidney disease and end-stage kidney disease (ESKD). Nowadays, there is consensus of opinion that an age over 60–65 years is not a contraindication to kidney biopsy (KB) [5, 6] but the level of evidence for patients over 80–85 years of age remains low. There is a general reluctance [7] to perform KB in very elderly patients due to the risk of complications (mostly bleeding) but these patients may also take full advantage of optimized therapeutic strategies [8–11].

To evaluate the risk/benefit balance of KB in very elderly patients, we analyzed the indications, adverse events and therapeutic impact of KB in a cohort of consecutive patients over 85 years of age.

## Materials and methods

In this retrospective multicenter cohort study, we analyzed the medical charts of all patients aged ≥85 years who underwent a native kidney biopsy (KB) in 9 nephrology centers from the Aix-Marseille area between 2010 and 2018. The same pathologists (LD and JT), from the Laboratory of Pathology of the University Hospitals of Marseille (AP-HM), are in charge of all KB in this area (550 native KB each year), which allowed the screening of all cases.

All the procedures followed were in accordance with the Helsinki Declaration of 1975, as revised in 2000.

The French authority on data protection “Commission Nationale Informatique et Libertés” (CNIL) authorized data collection for this study (authorization number: 2211854).

For each case, clinical information was collected from the medical chart with the help of the referring nephrologist. Recorded data included:

- age, gender, history of hypertension, diabetes, progressive cancer or cardiovascular disease
- physical examination: blood pressure, edema, body mass index, extra-renal symptoms
- laboratory values: serum creatinine, estimated glomerular filtration rate (eGFR) using the MDRD formula, serum albumin, urine protein/creatinine ratio, presence of hematuria
- main indication for KB
- pathological diagnosis
- KB-related adverse events
- therapeutic intervention after KB
- kidney function evolution and date of dialysis initiation if applicable
- date of the last follow-up and date of death if applicable.

The following immunological tests were collected when available: antinuclear antibodies, anti-double-stranded DNA antibodies, anti-neutrophilic cytoplasmic antibody, anti-phospholipase A2 receptor (PLA2R) antibodies, rheumatoid factor, cryoglobulinemia, complement C3 and C4, and serum and urine electrophoresis. Serological tests for hepatitis B, hepatitis C, syphilis and HIV were also collected.

Indications for KB were classified as: acute kidney injury (AKI), nephrotic syndrome (NS), coexisting AKI and NS, proteinuria without AKI, and chronic kidney disease (CKD).

AKI was defined, according to the KDIGO guidelines, as an increase in serum creatinine of at least 26.5 μmol/L within 48 h, or as an increase in serum creatinine to × 1.5 time baseline [12]. NS was defined as a proteinuria **≥**3.5 g/day with serum albumin < 3 g/dL. Significant proteinuria was defined as a urinary protein/creatinine ratio **≥** 0.5 g/g.

All KB were analyzed by the same renal pathologists (LD and JT), by light microscopy and immunofluorescence. For light microscopy, araldite-embedded sections were stained with Masson’s trichrome and Jones silver impregnation (2 and 0.2 μm sections respectively). For Immunofluorescence, 4 μm frozen sections were incubated with anti-Immunoglobulins, C3, and C1q antibodies (The binding site, 1/50 dilutions, Birmingham, UK).

Descriptive statistics are presented as medians and interquartile ranges for continuous variables and as absolute values and percentages for categorical variables. The study endpoints were overall survival (OS) and renal survival, computed from the date of the biopsy to the date of the event (all-cause death for OS; all-cause death or dialysis for renal survival) or censored at the date of the last follow-up in event-free patients. Median survival rates were estimated using the Kaplan-Meier method. Hazard ratio were estimated using Cox regression model. All factors associated with outcomes in the univariate analyses (*p*-value< 0.20) were fed into the multivariable models. All statistical tests were two-sided and the threshold for statistical significance was *p* < 0.05. Analyses were performed using IBM SPSS Statistics 20.0 (IBM Inc., New York, USA).

## Results

Between January 2010 and December 2018, a total of 114 very elderly patients underwent a native KB analyzed at the Laboratory of Pathology of AP-HM, which represents 2% of all native KB analyzed during the same period. Clinical data collection was insufficient for 10 patients, and 104 patients were included in this study. Follow-up data was available for 96 patients, who were considered in the survival analyses. The baseline characteristics of the study population are shown in Table 1.

**Table 1:** Baseline characteristics of the elderly patients (aged ≥85 years) included in this cohort. (Values are expressed as median [IQR] or number (%))

	*N* = 104
Age, years	87 [86–89]
Male	64 (61.5)
BMI, kg/m2	25 [21–28]
Hypertension	70 (67.3)
Diabetes mellitus	24 (23.1)
Progressive cancer	26 (25.0)
Cardiovascular disease	66 (63.5)
Serum creatinine, μmol/L	262 [159–416]
eGFR, ml/min/1.73 m2	16 [8–27]
Dialysis requirement	22 (21.2)
UPCR, g/g	1.4 [0.6–4.00]
Hematuria	43 (41.3)
Serum albumin, g/dL	28 [21–52.5]

(BMI, body mass index; eGFR, estimated glomerular filtration rate; UPCR Urinary protein to creatinine ratio)

**Fig. 1:**
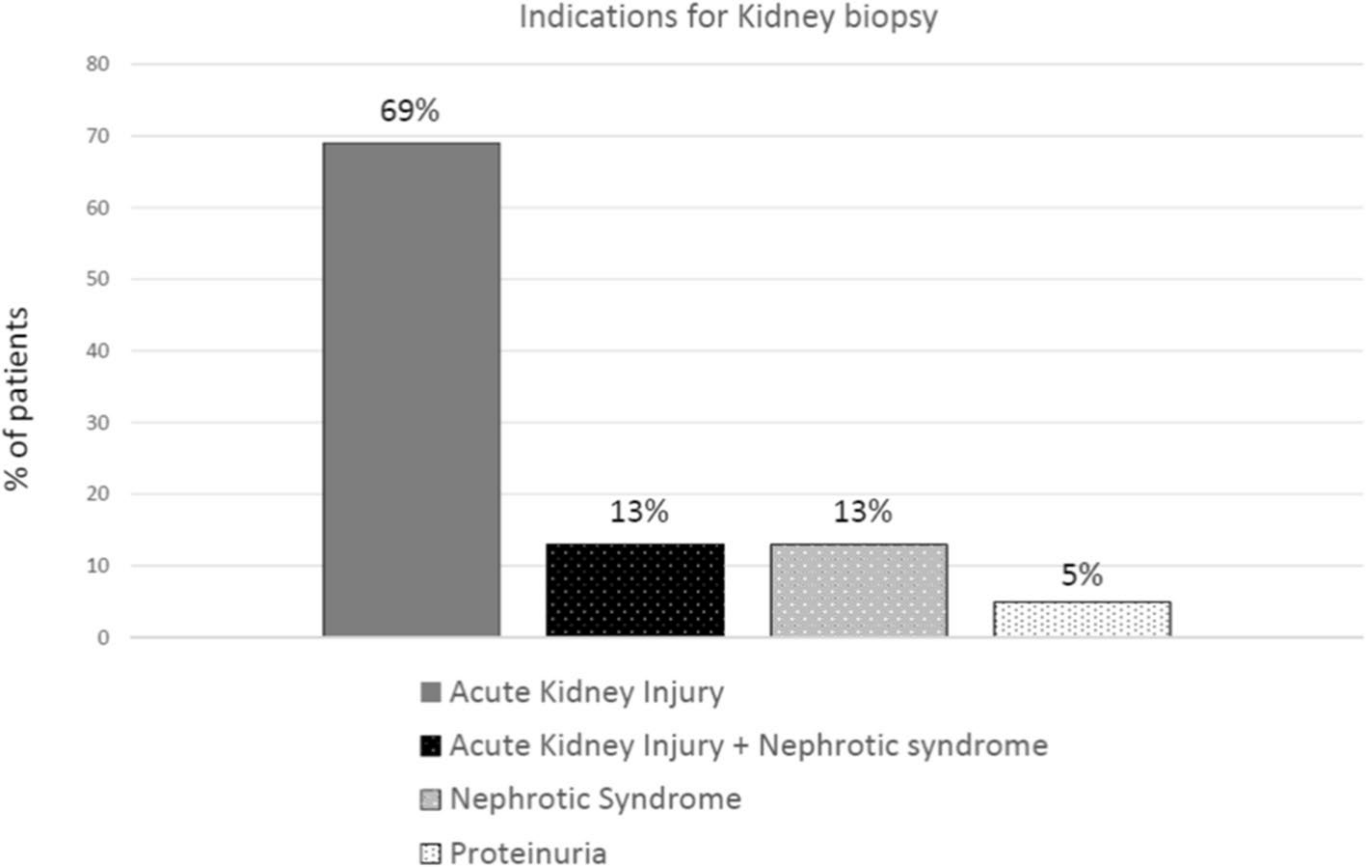
Indication for kidney biopsy in the 104 very elderly patients. (AKI, acute kidney injury; NS, nephrotic syndrome; PU, proteinuria)

The KB was performed during the course of a hospital stay in 73 patients, while 31 patients were hospitalized specifically for KB. The indications for KB are shown in Fig. 1. AKI was the most common indication (69.2% of patients), the other indications were NS with AKI (13.5%), NS without AKI (12.5%) and proteinuria (4.8%). No patient had a KB for the exploration of CKD alone. Significant proteinuria was present in 87.5% of patients, abnormal immunological tests in 27%, and extra-renal symptoms (mostly cutaneous lesions) in 17% of patients.

KB-related adverse events occurred in 7/104 (6.7, 95%CI 2.8–13.4) patients and are detailed in Table 2. Four (3.8%) patients required blood transfusion, including one patient for whom a radiological embolization was performed. No death was related to KB.

**Table 2:** Kidney biopsy-related adverse events, and therapeutic impact of kidney biopsy

	n (%)
**KB-related adverse events**	**7 (6.7)**
Mild bleeding or lumbar pain	3 (2.9)
Bleeding requiring blood transfusion	3 (2.9)
Bleeding requiring blood transfusion and radiological embolization	1 (0.9)
**Therapeutic intervention after KB**	**51 (49.0)**
corticosteroids	43 (41.3)
IV cyclophosphamide	7 (6.7)
rituximab	7 (6.7)
bortezomib	4 (3.8)
Other chemotherapy	4 (3.8)

Pathological results are displayed in Table 3. Non-diabetic glomerular diseases were diagnosed in 31 (29.8%) patients, including 10 (9.6%) with pauci-immune crescentic glomerulonephritis. Hypertensive nephropathy was diagnosed in 29 (27.9%) patients, and was the most frequent diagnostic in patients with proteinuria alone (4/5), as well as in patients with AKI (21/72) and in patients with NS and AKI (4/14). The most frequent diagnoses in patients with NS without AKI were membranous nephropathy (4/13 patients) and amyloidosis (AL or AA, 4/13 patients).

**Table 3:** Pathological diagnoses in the 104 native kidney biopsies

	n	(%)
**Vascular diseases**	**33**	**(31.7)**
Hypertensive nephropathy	29	(27.9)
Cholesterol embolism	2	(1.9)
Renal Infarction	1	(1)
Scleroderma renal crisis	1	(1)
**Tubulo-Interstitial diseases**	**25**	**(24)**
Acute interstitial nephritis	17	(16.3)
Acute tubular necrosis	7	(6.7)
Nephrocalcinosis	1	(1)
**Glomerular diseases**	**31**	**(29.8)**
Pauci immune crescentic GN	10	(9.6)
Membranous nephropathy	6	(5.8)
IgA nephropathy	6	(5.8)
embrano-proliferative GN	3	(2.8)
Minimal change disease	2	(1.9)
Focal segmental glomerular sclerosis	2	(1.9)
AA amyloidosis	2	(1.9)
**Hematological diseases**	**9**	**(8.7)**
AL amyloidosis	3	(2.8)
Light chain deposition disease	1	(1)
Myeloma cast nephropathy	2	(1.9)
Light chain tubular inclusions (Fanconi)	1	(1)
Leukemia (CMML) with blastic infiltration	1	(1)
Lymphomatous infiltration (NHL)	1	(1)
**Diabetic kidney disease**	**3**	**(2.9)**
**Non-contributive biopsy**	**3**	**(2.9)**

*GN* Glomerulonephritis; *CMML* Chronic myelomonocytic leukemia; *NHL* Non-Hodgkin lymphoma

Overall, a disease likely to respond to a specific treatment was identified in 61 patients (58.7, 95%CI 48.6–68.2%): 56.9% (41/72) of patients with AKI, 71.4% (10/14) of patients with NS and AKI, 69.2% (9/13) of patients with NS without AKI, and 20% (1/5) of patients with proteinuria.

A specific therapy was administered to 51 patients (49, 95%CI 39.1–59.0%) following KB (Table 2), among whom: 6 patients with IgA nephropathy (including 3 Henoch-Schönlein purpura), 4 patients with minimal change disease or focal segmental glomerulosclerosis, and 13 patients with acute interstitial nephritis received corticosteroids; 9 patients with pauci-immune crescentic glomerulonephritis were treated with corticosteroids, associated with cyclophosphamide in 7 and with rituximab in 2; 3 patients with membranous nephropathy received rituximab; 1 patient received rituximab for B lymphoma with AL amyloidosis, and another 1 for membrano-proliferative GN; 4 patients received corticosteroids and bortezomib for AL amyloidosis (*n* = 1), myeloma cast nephropathy (*n* = 2) or monoclonal Ig light chain-associated Fanconi disease (*n* = 1).

**Fig. 2:**
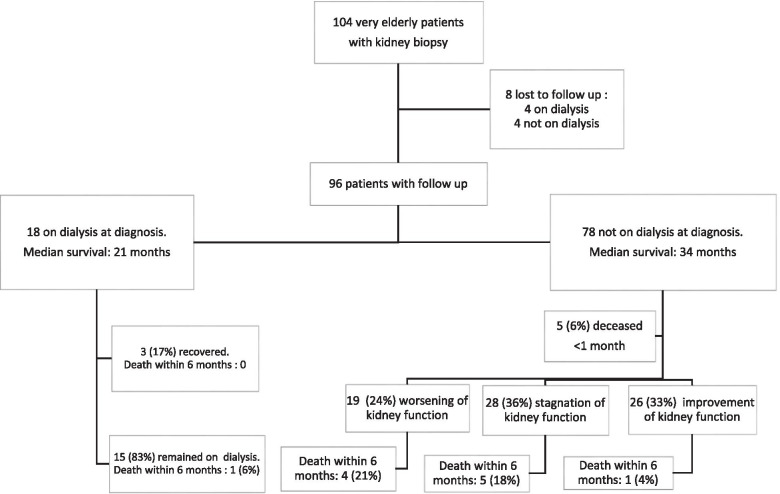
Patient outcome

Follow-up data were available for 96 patients (8 were lost to follow-up), their outcomes are displayed in Fig. 2. Median follow-up was 17 months (IQR 7–34 months), and follow-up ranged from 10 days (death of the patient) to 97 months. Sixteen (17%) patients died within 6 months after KB. Among the 18 patients who were depending on dialysis at the time of KB, 3 recovered and 1 died within 6 months. Among the 78 patients who were not on dialysis at the time of KB, 5 died within a month, 19 had a worsening of kidney function, 26 an improvement of kidney function, and 28 remained stable; 10 additional patients died within 6 months.

**Fig. 3:**
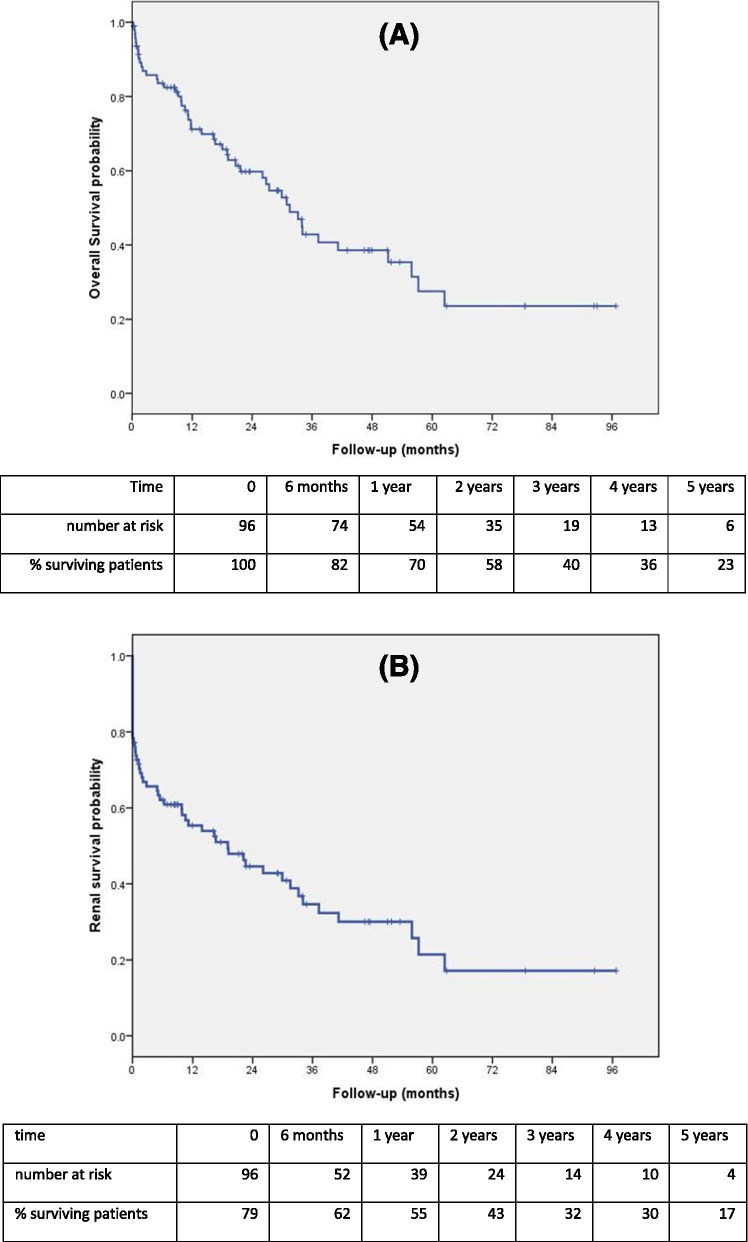
Kaplan Meier survival analyses. **A**) Overall survival. **B**) Renal survival

Overall survival is shown in Fig. 3A. Median overall survival was 32 months. Renal survival (composite survival: no death and no dialysis) is shown in Fig. 3B. Median renal survival was 19 months. Factors associated with overall and renal survival, by univariate and multivariate analyses, are detailed in Table 1. Bleeding complications of KB and baseline cardiovascular disease were independently associated with impaired overall and renal survival. In addition, vascular kidney disease on KB and baseline eGFR < 15 mL/min/1.73m^2^ were independent predictors of impaired renal survival.

**Table 4:** Factors associated with overall survival and renal survival

	Univariate analysis	Univariate analysis				Multivariable analysis			
	Variable	Hazard ratio	95%CI inf	95%CI sup	***p***-value	Hazard ratio	95%CI inf	95%CI sup	*p*-value
***Overall Survival***	Gender Male	1.1	0.6	2.0	0.701				
	Indication for KB: acute kidney injury	1.7	0.7	4.4	0.243				
	Absence of specific treatment				0.360				
	Specific treatment other than corticosteroids	1.6	0.6	4.3	0.323				
	Specific treatment with corticosteroids	0.8	0.4	1.5	0.467				
	Age above 87 years	1.0	0.6	1.8	0.958				
	Acute kidney injury	1.9	0.6	6.2	0.271				
	Nephrotic syndrome	1.0	0.6	1.9	0.882				
	Proteinuria level	0.9	0.5	1.7	0.755				
	Glomerular kidney disease	0.6	0.3	1.2	0.128	NS			
	Tubulo-interstitial kidney disease	0.8	0.4	1.7	0.634				
	Vascular kidney disease	1.5	0.8	2.6	0.206				
	eGFR < 15 ml/min/1.73 m2 at kidney biopsy	1.6	0.9	2.9	0.106	NS			
	eGFR < 30 ml/min/1.73 m2 at kidney biopsy	1.9	0.9	4.2	0.091	NS			
	Body mass index < 20 kg/m2	1.0	0.3	3.3	0.982				
	Serum albumin < 3 g/dL	0.9	0.4	1.8	0.732				
	Haematuria	1.3	0.7	2.3	0.358				
	Hospitalized specifically for kidney biopsy	0.7	0.4	1.4	0.361				
	**Bleeding complications of kidney biopsy**	2.8	1.1	7.3	**0.034**	3.2	1.2	8.2	**0.019**
	Diabetes	0.8	0.4	1.6	0.529				
	Hypertension	0.6	0.3	1.1	0.109	NS			
	Current malignancy	1.2	0.6	2.3	0.657				
	**Cardiovascular disease**	2.1	1.1	4.2	**0.029**	2.2	1.1	4.3	**0.024**
***Renal Survival***	Gender Male	1.4	0.8	2.4	0.281				
	Indication for KB: acute kidney injury	4.2	1.3	13.5	**0.016**	NS			
	Absence of specific treatment				0.150	NS			
	Specific treatment other than corticosteroids	1.2	0.5	2.9	0.700				
	Specific treatment with corticosteroids	0.6	0.3	1.1	0.084	NS			
	Age above 87 years	0.9	0.5	1.5	0.641				
	Acute kidney injury	3.0	0.9	9.7	0.061	NS			
	Nephrotic syndrome	0.7	0.4	1.4	0.336				
	Proteinuria level	0.7	0.4	1.3	0.329				
	Glomerular kidney disease	0.5	0.3	1.0	**0.035**	NS			
	Tubulo-interstitial kidney disease	1.1	0.6	2.0	0.731				
	**Vascular kidney disease**	1.6	0.9	2.8	0.085	2.1	1.1	3.8	**0.020**
	**eGFR < 15 ml/min/1.73 m2 at kidney biopsy**	2.3	1.4	4.0	**0.002**	2.4	1.3	4.3	**0.005**
	eGFR < 30 ml/min/1.73 m2 at kidney biopsy	1.8	0.9	3.7	0.080	NS			
	Body mass index < 20 kg/m2	1.3	0.4	4.4	0.655				
	Serum albumin < 3 g/dL	0.8	0.4	1.5	0.442				
	Haematuria	0.9	0.5	1.6	0.764				
	Hospitalized specifically for kidney biopsy	0.6	0.3	1.1	0.111	NS			
	**Bleeding complications of kidney biopsy**	4.4	1.9	10.2	0.001	4.5	1.9	10.8	**< 0.001**
	Diabetes	0.7	0.3	1.3	0.205				
	Hypertension	0.7	0.4	1.2	0.146	NS			
	Current malignancy	1.1	0.6	2.0	0.713				
	**Cardiovascular disease**	2.5	1.3	4.6	**0.005**	2.1	1.1	3.9	**0.025**

*NS* Not significant in multivariable analysis

## Discussion

We report here a large cohort of very elderly patients who underwent a native KB and show that the adverse events were not frequent and that the result of KB led to a specific therapeutic strategy in half of patients. In patients for whom no specific treatment was indicated, KB may have prevented the initiation of a potentially harmful treatment (such as corticosteroids). This work shows that for some patients, even if they are very old, the KB remains a useful procedure with therapeutic implications, especially in case of AKI and/or NS.

In the present cohort, the bleeding risk of KB was significant but not prohibitive (3% requiring transfusion), without KB-related death. The same level of complication was identified in other series of elderly patients [13]. Even if lower rates of adverse events were reported in younger populations [14], several studies from the literature comparing the bleeding risks of KB in patients of different ages showed bleeding rates similar to the present cohort [15, 16]. In previous studies, the baseline serum creatinine level has been reported to predict bleeding complications after KB [15]. Indeed, in the present cohort, all the patients with bleeding complications displayed AKI, and 3/7 were on dialysis. Halimi et al. [17], in a large national French retrospective cohort study published in 2020, reported a major bleeding after native KB in 2765 of 52,138 (5%) patients (blood transfusions: 5%; angiographic intervention: 0.4%; and nephrectomy: 0.1%). Independent predictors of bleeding in this cohort were gender, frailty index, anemia, and altered kidney function. Advanced age was not an independent risk factor for bleeding in this large cohort.

Although patients were not selected in this retrospective study, an indication bias is likely in these very elderly subjects, as clinicians may have refrained from performing a KB in the frailest patients or in patients with mild kidney involvement. This may explain why a large majority of patients from this cohort displayed AKI. The question of the futility of performing an invasive procedure such as KB in very old patients, whose life expectancy is probably limited, may arise. Yet, although their median age was 87 years, only 16.7% (16/96) of patients died within 6 months after KB. For comparison purposes, the 1-year survival rate for incident patients on dialysis is 68% in France [4].

The diagnostic benefit of KB in the very elderly has been reported before [18, 19]. Moutzouris et al. [20] in an American cohort of 235 patients over 80 years of age, reported a therapeutic impact of KB in 67% of patients, particularly in those with AKI or NS. In the present cohort, the therapeutic impact of KB also depended on the indication for KB, patients with NS and/or AKI being the most likely to display a treatable condition, compared to patients with proteinuria alone.

AKI is associated with increased morbidity and mortality, especially in elderly subjects: the in-hospital mortality of elderly patients with AKI ranges from 15 to 40% [20–24]. Pre-renal AKI, essentially due to volume depletion, and obstructive renal failure are the most common cause of AKI in elderly patients [23]. Shock, nephrotoxic drugs and rhabdomyolysis are also frequent causes of AKI. But when the clinical context is far from obvious, or when a glomerulonephritis is suspected, a KB may be useful even in elderly patients to guide the therapeutic strategy [24]. In the present cohort, as in other European cohorts [19, 25], AKI was the most common indication for KB in elderly patients. On the contrary, NS was the main indication for KB in different series of elderly patients from Asia (50% of patients aged **≥**80 in Japan [26] 69% of patients aged > 80 years in China [6]).

Acute interstitial nephritis (AIN) was the second most frequent diagnosis after hypertensive nephropathy in this cohort. Most AIN were immuno-allergic, related to the prescription of proton pump inhibitors, vitamin K antagonist or antibiotics, which are often prescribed in the elderly. In this condition, KB allows the withdrawal of the drug, and sometimes the prescription of corticosteroids.

Pauci-immune crescentic GN was the third most frequent diagnosis in this cohort. A high frequency of crescentic GN in elderly patients was also reported in several studies from different countries (10% here, 14% in Spain [25] 11% in Japan [26] 13% in Italy [27]). KB is not always required for the diagnostic of ANCA-associated vasculitis. Yet, it can confirm the diagnosis in patients without ANCA or with atypical clinical presentation and indicate the presence of active proliferative lesions likely to benefit from immunosuppressive treatment. Indeed, immunosuppressive therapy can improve kidney function and systemic disease in ANCA-associated vasculitis, even in elderly patients [28]. In an American cohort of 78 patients over 80 years of age with biopsy-proven pauci-immune GN published in 2011 [9], the use of immunosuppressive therapy was associated with significantly lower risk of ESKD, death, and the combined outcome of ESKD or death.

KB can also be valuable for the diagnosis of haematological malignancies. In particular, AL amyloidosis without myeloma is not always detected on extra-renal tissue (such as minor salivary glands), and the detection of light chain amyloid deposits in a patient with NS can lead to the initiation of a chemotherapy which was not indicated on haematological parameters only. This was the case for 5 patients with hematological diseases from the present cohort.

There are some limitations to this study, in addition to its retrospective design. First, no data was available on the evaluation of frailty, autonomy and quality of life of the elderly patients included here. Second, no data was gathered on the clinical characteristics and outcomes of elderly patients with AKI and/or NS who were not proposed a KB. This hampers the evaluation of the “right indications for KB” and of the benefit of KB in the global population of elderly patients.

Yet, the focus of this work on very elderly patients (≥85 years), and the comprehensive analysis of KB indications, results, and therapeutic impact, together with the information on patients’ outcomes, brings new information to the field.

In conclusion, this study provides detailed information on the benefits and risks of KB in a population of very elderly patients in France. The reasonable bleeding risk and the high proportion of pathological diagnoses with a therapeutic impact, especially in patients with AKI and/or NS, pleads for the interest of this invasive procedure in some very elderly patients, irrespective of their age.

## Data Availability

The datasets used and analysed during the current study available from the corresponding author on reasonable request.

## Abbreviation

AKI: Acute kidney injury
AIN: Acute interstitial nephritis
BMI: Body mass index
CKD: Chronic kidney disease
CMML: Chronic myelomonocytic leukemia
ESKD: End-stage kidney disease
eGFR: Estimated glomerular filtration rate
GN: Glomerulonephritis
KB: Kidney biopsy
NHL: Non-hodgkin lymphoma
NS: Nephrotic syndrome
OS: Overall survival
PU: Proteinuria
UPCR: Urinary protein to creatinine ratio
